# Can Intensified Tuberculosis Case Finding Efforts at Nutrition Rehabilitation Centers Lead to Pediatric Case Detection in Bihar, India?

**DOI:** 10.4236/jtr.2016.41006

**Published:** 2016-03-30

**Authors:** Rajeev R. Pathak, Bal Krishna Mishra, Patrick K. Moonan, Sreenivas A. Nair, Ajay M. V. Kumar, Mohit P. Gandhi, Shamim Mannan, Smita Ghosh

**Affiliations:** 1 WHO Country Office for India, New Delhi, India; 2 State Tuberculosis Training and Demonstration Centre, Patna, India; 3 US Centers for Disease Control and Prevention, Division of TB Elimination, Atlanta, USA; 4 International Union Against Tuberculosis and Lung Disease (The Union), South-East Asia Regional Office, New Delhi, India

**Keywords:** Tuberculosis, Children, Malnutrition, India, Screening

## Abstract

**Introduction:**

Seven district-level Nutritional Rehabilitation Centres (NRCs) in Bihar, India provide clinical and nutritional care for children with severe acute malnutrition (SAM).

**Aim:**

To assess whether intensified case finding (ICF) strategies at NRCs can lead to pediatric case detection among SAM children and link them to TB treatment under the Revised National Tuberculosis Control Programme (RNTCP).

**Materials and Methods:**

A retrospective cohort study was conducted that included medical record reviews of SAM children registered for TB screening and RNTCP care during July–December 2012.

**Results:**

Among 440 SAM children screened, 39 (8.8%) were diagnosed with TB. Among these, 34 (87%) initiated TB treatment and 18 (53%) were registered with the RNTCP. Of 16 children not registered under the RNTCP, nine (56%) weighed below six kilograms—the current weight requirement for receiving drugs under RNTCP.

**Conclusion:**

ICF approaches are feasible at NRCs; however, screening for TB entails diagnostic challenges, especially among SAM children. However, only half of the children diagnosed with TB were treated by the RNTCP. More effort is needed to link this vulnerable population to TB services in addition to introducing child-friendly drug formulations for covering children weighing less than six kilograms.

## 1. Introduction

Of the 9 million annual tuberculosis (TB) cases, about 1 million (11%) occur in children (under 15 years of age) [1]. Of these childhood cases, 75% occur annually in 22 high-burden countries that account for 80% of the world’s estimated incident cases [1]. Young children are at increased risk of rapid progression from infection to disease, reflecting recent transmission rather than secondary reactivation [1] [2]. Thus, increased case detection of tuberculosis (TB) using active or intensified case finding (ICF), taking into consideration the particular epidemiology and clinical presentation of TB in children, is an important STOP TB strategy [2]–[5].

Risk factors for TB in children include 1) age less than 5 years; 2) close/household contacts with a case of pulmonary TB (especially smear-positive or culture-positive pulmonary TB); 3) HIV infection, and 4) severe malnutrition [1]–[3].

Severe malnutrition is a key risk factor in high-TB burden settings and plays an important role in cell-mediated immunity, the key host defense against TB [6] [7]. However, to our knowledge, very few studies have explored the feasibility of ICF among malnourished children in Nutrition Rehabilitation Centers (NRC) [8]. One of the 10 steps in the WHO’s “Roadmap for childhood TB: Steps towards zero deaths” specifies not missing critical opportunities for intervention [2]. Another step outlined in the same document recommends developing community-centered strategies for children [2].

India has one of the highest burdens of both malnutrition and TB among children in the world [9]–[11]. Approximately 9 million children with severe acute malnutrition (SAM) live in India [12]. India also accounts for 16% of all childhood TB cases worldwide [10]–[12]. The high-burden states of Bihar, Madhya Pradesh, Uttar Pradesh and Rajasthan account for 43% of all malnourished and 38% of all TB among children in India [11] [12].

In response to an increased number of SAM children and malnutrition-related deaths in Bihar [12], the Government of India’s National Rural Health Mission established Nutritional Rehabilitation Centres (NRCs) in 2011. NRC paediatricians clinically manage SAM children through therapeutic feeding and growth monitoring. They also provide immunizations, diagnose and treat infectious diseases, including TB. Historically, India’s Revised National Tuberculosis Control Programme (RNTCP) has primarily focused on adults, using sputum-smear microscopy for diagnosis, a tool that is not as effective for children with tuberculosis [13] [14]. In 2012, the RNTCP and Indian Academy of Pediatrics (IAP) developed a consensus statement for standardized diagnosis and management for childhood tuberculosis [15] [16]. The feasibility of applying the national diagnostic algorithm has not been fully evaluated among children with malnutrition. Intensified case finding at NRCs may provide an opportunity to identify and link children with TB to care. Thus, we sought to determine the proportion of TB cases detected among the eligible SAM children screened according to the national diagnostic algorithm at NRCs and documented the proportion that was registered with the RNTCP for TB treatment and care.

## 2. Methodology

### 2.1. Study Setting

Bihar is the third most populated state in India (103.8 million persons), including 18.6 million (18%) children aged five years or younger [17]. NRCs in Bihar are staffed by medical officers and nurses who provide medical and nutritional support according to national and international guidelines for management of severe malnutrition and child growth standards [18] [19]. NRCs follow an admission cycle for treating a cohort of 20 new children (aged 6 – 60 months old) every 21 days.

### 2.2. Study Design

At 7 conveniently selected NRCs, SAM children were evaluated according to the national TB diagnostic algorithm. Briefly, this algorithm leads to TB diagnosis, if a child has: 1) persistent cough or (unspecified) fever for more than 14 days; or 2) history of unexplained weight loss (*i.e.*, loss of more than 5% body weight as compared to highest weight recorded in last 3 months) or no weight gain in past 3 months; or 3) history of exposure to a sputum-smear microscopy positive TB case, followed by either a positive sputum-smear microscopy result or a combination of Mantoux tuberculin skin test (TST) of ≥10 mm and chest radiograph suggestive of TB [15] [16]. Children screened and diagnosed with TB at a NRC were initiated on treatment and reported to the RNTCP. As per RNTCP guidelines and due to the current weight requirement for RNTCP TB regimens [15] [16], persons equal to 6.0 Kg body weight or above were eligible for enrolment and TB treatment by RNTCP, whereas persons under 6.0 Kg body weight are referred to private providers for treatment. SAM children were weighed daily to achieve a target increased body weight gain of 15% during the 21 days of inpatient care at the NRC. A female attendant, preferably the mother, stayed with the child and was counseled and educated about nutritional supplementation and was compensated for any potential wages lost [19].

### 2.3. Data Collection

This observational study included routinely collected programme data abstracted from NRC medical records and RNTCP registers and treatment logs at seven conveniently selected, district-level NRCs in Bihar, India during July–December 2012. For children who received 21-days of inpatient treatment at NRC, we abstracted basic demographic and clinical information (*i.e.*, sex, age, body weight, rural or urban settings, and TB diagnosis information) from individual medical records using standardized data collection instruments.

### 2.4. Data Management and Analysis

Patient names and date of birth in the NRC admission logs were manually cross-matched to sub-district (*i.e*., Tuberculosis Unit) level TB registers. EpiData (version 3.1 for entry and 2.2.2.182 for analysis, EpiData Association, Odense, Denmark) was used for double-data entry, data validation and to generate descriptive statistics. We reported case counts and proportions of select demographic characteristics amongst children that were screened and diagnosed with TB according to the IAP diagnostic algorithm and the proportion that were linked to RNTCP for treatment and care [15] [16]. Chi square statistics were generated to study statistical associations of a screening method with diagnosing TB vs not.

### 2.5. Ethics Consideration

This study was reviewed and approved by the Institutional Ethics Committee of the National Tuberculosis Institute (Bangalore, India) and the Ethics Advisory Group of the International Union Against Tuberculosis and Lung Disease (Paris, France). Since data collected were part of routine TB control efforts, individual patient consent or parental assent was not required. Participation of U.S. Centers for Disease Control and Prevention (CDC) in this project did not meet the definition of engagement in human subjects’ research because the CDC investigators did not interact with study subjects or have access to patient identifiable data, thus a separate institutional review board approval was not required.

## 3. Results

**Figure 1:**
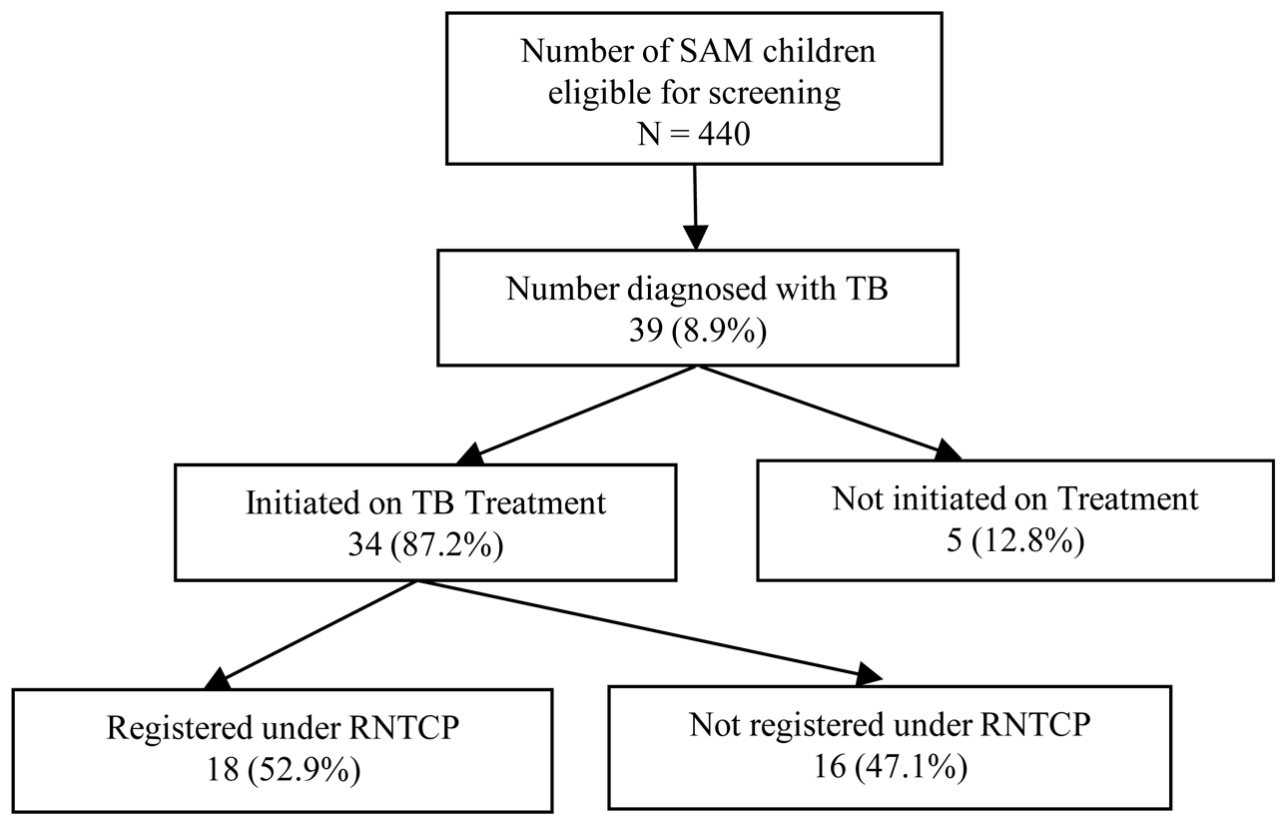
TB screening of SAM children eligible for screening based upon the national diagnostic algorithm for pediatric tuberculosis at NRCs of Bihar, India.

During July–December, 2012, a total of 440 SAM children were admitted to the district-level NRCs. Despite all SAM children qualifying for TB screening, due to their history of unexplained weight loss or no weight gain in past 3 months, only 68% (n = 301) were screened by TST, 14% (n = 61) by chest X-ray, and less than 1% (n = 3) by sputum smear microscopy. A total of 39 (8.8%) children were diagnosed with TB (pulmonary, n = 32; extra-pulmonary, n = 7), among these, 34 (87%) were initiated on antituberculosis drug treatment, but only 18 (53%) were linked to follow-up and care with the RNTCP (Figure 1). Except for sputum microscopy, all other screening methods were significantly associated with the children being diagnosed with TB vs. not (Table 1). Among the 32 children diagnosed with pulmonary TB, 17 (53%) had both TST positive and CXR suggestive of TB disease (Table 1). Thirty-six (92%) children had a history of persistent cough for more than 14 days and 22 (56%) had a history of contact with a TB case (Table 1). Among the 7 extra-pulmonary TB cases, 2 (29%) were diagnosed clinically because of prolonged history of cough and contact with a TB case.

Of the children who did not initiate TB treatment (n = 5), 2 (40%) were transferred to tertiary care centres due to SAM-related clinical complications and we do not know if they started or completed TB treatment. We were unable to ascertain and document the reason TB treatment was not initiated for the remaining 3 children.

Despite more females than males being treated at these NRCs (61% vs. 39%), more males were diagnosed with TB than females (10% vs. 8%). More children aged 12 – 35 months were diagnosed with TB than other age groups, accounting for 69% of all cases evaluated and 24 out of 28 (86%) initiated on treatment (Table 2). Out of 16 SAM children not enrolled in RNTCP after discharge from NRCs, 9 (56%) were less than 6.0 Kg body weight, a criteria for receiving treatment within the programme.

## 4. Discussion

All of the children included in this analysis were by definition malnourished, characterized by nonspecific weight loss or failure to gain weight prior to TB screening, one of three qualifying clinical indications for additional TB diagnostics (*i.e.*, sputum-smear microscopy or a combination of chest radiograph and Mantoux TST) according to the national pediatric TB diagnostic algorithm; however few were screened by sputum smear, X-ray, or by TST (1%, 17%, 68%, respectively). This may indicate under-diagnosis of both pulmonary and extra-pulmonary forms of TB.

There are opportunities for improvement in these screening practices, especially in high-risk population centres like NRCs. Despite strong language offered by the joint RNTCP/IAP consensus statement, which emphasizes sputum smear microscopy as one of the primary tools for accurate TB diagnosis including aggressive collection of alternative specimens (e.g., gastric lavage, induced sputum, bronchoalveolar lavage) [20] [21], very few NRC clinicians followed these guidelines. In practice, most childhood cases cannot be confirmed through sputum (*i.e*., smear-negative) and alternate sources of specimen collection through gastric aspirates or cerebral spinal fluids (CSF) in non-specialized settings such as NRCs are not feasible [22] [23]. The diversity of relatively nonspecific clinical presentations, an immature immune system, the paucibacillary nature of childhood TB, and the difficulty for young children in producing adequate sputum on demand all add to this diagnostic challenge [14]. These challenges were reflected by the clinicians in our study, where only 3 (0.7%) children received a sputum examination. This may be explained by the well-documented poor performance of sputum smear for diagnosis of TB in children, but also the effects of severe malnutrition upon immature immune systems [6].

Screening and diagnosing TB in children can be challenging. We hypothesize several reasons for the NRC clinicians’ inability to follow the diagnostic algorithm. First, despite having a medical officer at all participating NRCs, only 3 (43%) employed a qualified paediatrician. Second, chest radiography was not available on site at 4 (57%) of the NRCs. Radiography was thus outsourced to private providers and their use was often limited to children with respiratory symptoms. Even when radiographs were available, in a similar way to sputum-smear microscopy, there were logistical and diagnostic challenges for the identification of TB in children from radiologic images. Depending on the degree of disease progression and the immunological composition of the child, interpreting a roentgenogram may be difficult without considerable training and experience. Children rarely develop cavitary lesions, the hallmark of pulmonary TB in adults, but rather present with a variety of subtle radiological manifestations, such as endobronchial disease or lymphadenopathy [24]. Third, during the initial three months of the study period, tuberculin was unavailable at all NRCs and thus TST was outsourced to external generalized diagnostic centres, which may have lacked expertise in following national guidelines for administering TST and its interpretation. TST may supplement the diagnosis of pediatric TB, especially among bacteriologically unconfirmed cases. However, as malnutrition is known to directly impact cell-mediated immunity [6] [7], it was possible that some SAM children were unable to demonstrate a sufficient immune response to TST [7] [25] [26]. Therefore, negative TST results alone are not sufficient to rule-out TB infection or active TB disease in this population [14] [26]. Finally, as outlined above, sputum collection is a major challenge in the diagnosis and confirmation of childhood TB; very few children had specimens collected. GeneXpert^®^ MTB/RIF (Cepheid, Sunnyvale, CA, USA), now more widely available in India, could be used rather than conventional microscopy, as the initial test in all children suspected of having TB [27] [28]. For the above challenges described, it was possible that some cases of TB were missed, especially among the vast majority who did not undergo the full diagnostic algorithm (Table 1).

Despite the challenges and potential barriers to accurate diagnosis, screening for TB at NRCs identified 39 cases. In other words, for every 11 SAM children screened, 1 TB case was detected. Or in population context, the equivalent of 89 TB cases per 1000 children screened; emphasizing that such screening of this vulnerable population may have a potential to be a high-yield activity [5]. By comparison, other widely recognized TB screening activities such as screening at HIV clinics or as part of TB contact investigation require more persons screened to detect one case (58 and 34 persons, respectively) [5]. These results should be interpreted with caution, however. Our study population was derived from a convenience sample of NRCs and may not be representative of all SAM children in India.

Another study of TB case finding among SAM children in India has shown a similar TB case evaluation rate of 72% [8]. However, detecting cases alone will not reduce TB morbidity and mortality in a community. Case detection must be linked to adequate antituberculosis drug treatment and management for at least 6 months. We found that only 18 (53%) children with TB were enrolled in RNTCP for treatment and follow up after discharge from NRCs. Nine out of the 16 (47%) not enrolled in RNTCP were under 6.0 Kg in body weight. Currently, the RNTCP does not offer a child-friendly drug formulation for weight bands less than 6.0 Kg. We hypothesize this could be a major factor for low reporting to RNTCP and linkage to care and follow up, though this was not directly explored.

Over half of the SAM children with a TB diagnosis [n = 27, (56%)] had known exposure to a sputum-smear positive case of TB (Table 1). This presents an additional opportunity for active case finding and preventing further transmission in the community. While not standard practice in India, source-case investigations have been shown to be effective in low-incidence settings for children with both LTBI and active TB disease [23] [29]–[31]. Even though childhood TB is a sentinel event representing recent transmission [31], it is unclear if an approach for source case investigations and treatment of LTBI would be cost-effective in a high TB burden country like India.

This study had several major limitations. The study sample was not representative; sites were chosen on a basis of convenience due to administrative and financial challenges, and we have no data to show whether the group studied is similar or different from the larger universe of SAM children in all 32 NRCs in Bihar or other high-TB burden states that have NRCs. Since our data were based on abstraction of routinely collected data, as is the case with all data abstraction studies of routine surveillance and medical records, data may be of limited quality. Surveillance registries and medical records were not designed for study purposes, but rather to document clinical encounters and report illness of public health importance. It is possible that some data errors occurred while transcribing data across NRC and RNTCP registers.

## 5. Conclusion

Screening for TB at NRCs appears to be a high-yield activity. Expansion of such screening activities for high-risk children at other NRCs of Bihar or other states, could lead to detection of additional paediatric cases and LTBI. However, malnourished children present a complex diagnostic and clinical challenge. NRCs must ensure that these at-risk children are thoroughly assessed and ample resources are in place to both initiate and complete a full course of TB treatment. Further research is needed to better understand the sensitivity of the national diagnostic algorithm as it applies to malnourished children, especially amongst those with known exposure to a sputum-smear positive TB case. Development and evaluation of new diagnostics for children, especially in high-risk settings like NRCs are needed to better understand disease burden of this vulnerable population. Moreover, in an effort to provide universal access to free TB services in India, RNTCP should consider offering appropriate drug formulations irrespective of weight, including children less than 6 kilograms. Future availability of shorter, child-friendly regimens for both latent infection and active disease and use of improved diagnostics like GeneXpert^®^ MTB/RIF may facilitate intensive case finding, treatment, better reporting, and linkage to care.

## Figures and Tables

**Table 1:** Tuberculosis diagnosis at Nutrition Rehabilitation Centres (NRCs) among 440 Severely Acute Malnourished (SAM) children eligible for screening by diagnostic methods.

	SAM children with tuberculosis (TB) n (%)	SAM children without TB n (%)	Total SAM children eligible for screening N = 440	Chi square p-values
**Sputum smear microscopy**				
Positive	0 (0.0)	0 (0.0)	0	0.244
Negative	1 (2.6)	2 (0.5)	3	
Not done/not recorded	38 (97.4)	399 (99.5)	437	
**Tuberculin skin test > 10 mm**				
Yes	20 (51.3)	1 (0.2)	21	<0.001
No	10 (25.6)	270 (67.3)	280	
Not done/not recorded	9 (23.1)	130 (32.4)	139	
**Chest radiography**				
Suggestive of TB	29 (74.4)	0 (0.0)	29	<0.001
Not suggestive of TB	5 (12.8)	27 (6.7)	44	
Not recorded	5 (12.8)	374 (93.3)	391	
**History of cough**^a^				
Yes	36 (92.3)	40 (10.0)	76	<0.001
No	3 (7.7)	350 (87.3)	353	
Not recorded	0 (0.0)	11 (2.7)	11	
**Household exposure to a known TB case**				
Yes	22 (56.4)	40 (10.0)	62	<0.001
No	17 (43.6)	341 (85.0)	360	
Not recorded	0 (0.0)	18 (4.5)	18	

a History of cough for 14 days or more.

**Table 2:** Characteristics of children with Severe Acute Malnutrition (SAM), admitted from July to December 2012 in seven Nutritional Rehabilitation Centres (NRCs) in Bihar, India.

Characteristics	Number screened	TB cases diagnosed	Treatment initiated among diagnosed	Registered in RNTCP among those initiated on TB treatment

	n (%)^1^	n (%)^2^	n (%)^2^	n (%)^2^

	440 (100)	39 (9)	34 (87.2)	18 (52.9)
**Sex**				
Male	168 (38.2)	17 (10.1)	15 (88.2)	6 (40.0)
Female	272 (61.8)	22 (8.0)	19 (86.4)	12 (63.1)
**Age (in months)**				
6 – 11	78 (17.7)	5 (6.4)	4 (80.0)	0 (0.0)
12 – 35	310 (70.5)	28 (9.0)	24 (85.7)	14 (58.3)
36 – 60	52 (11.8)	6 (11.5)	6 (100.0)	4 (83.3)
**Settings**				
Rural	241 (54.8)	22 (9.1)	20 (90.9)	10 (50)
Urban	199 (45.2)	17 (8.5)	14 (82.3)	8 (57.1)
**Body weight**				
>6 Kg	241 (54.8)	25 (10.3)	24 (96.0)	17 (70.8)
<6 Kg	199 (45.2)	14 (7.0)	10 (71.4)	1 (10.0)

1 Column %;

2 Row %.
